# Microbiota-Associated Amino Acid Metabolites in Inflammatory Bowel Disease: Emerging Key Players in the Host–Microbe Interface

**DOI:** 10.3390/microorganisms14092108

**Published:** 2026-09-20

**Authors:** Xianwen Meng, Chunyang Tian, Yijun Zhu, Danping Zheng

**Affiliations:** 1 Department of Gastroenterology, The First Affiliated Hospital, Sun Yat-sen University, Guangzhou 510080, China; mengxw3@mail2.sysu.edu.cn (X.M.); tianchy5@mail2.sysu.edu.cn (C.T.); 2 Institute of Precision Medicine, The First Affiliated Hospital, Sun Yat-sen University, Guangzhou 510080, China; zhuyj67@mail.sysu.edu.cn

**Keywords:** gut microbiome, metabolites, amino acids, inflammatory bowel disease, host-microbiota interaction, metabolomics

## Abstract

Inflammatory bowel disease (IBD), encompassing Crohn’s disease (CD) and ulcerative colitis (UC), is a chronic relapsing intestinal inflammatory disorder driven by complex interactions among host genetics, immune dysregulation, and gut microbiota dysbiosis. The functional contribution of microbial metabolites beyond short-chain fatty acids and bile acids remains incompletely understood. A critical and unresolved question is whether microbiota-associated amino acid metabolites are merely passive indicators of dysbiosis or active drivers of intestinal inflammation and tissue repair. In this review, we summarize recent advances in the roles of microbiota-associated amino acid metabolites and their derivatives in IBD, highlighting that amino acid metabolites constitute a functionally distinct class of bioactive signaling molecules that operate through four representative metabolic networks: tryptophan metabolism, aspartate-related metabolism, branched-chain amino acid (BCAA) metabolism, and arginine–polyamine metabolism. We synthesize recent clinical metabolomic data and identify reductions in tryptophan-derived indoles, glutamate, histidine, and selected BCAAs across IBD clinical cohorts and sample matrices, whereas metabolites such as serine, proline, and polyamine degradation products are frequently elevated or associated with disease activity or therapeutic response. Mechanistically, microbiota-associated amino acid metabolites are implicated in intestinal homeostasis and IBD pathogenesis through multifaceted pathways, including modulation of intestinal epithelial barrier integrity (e.g., cell–cell junction, mucus secretion, and stem cell-driven mucosal repair), innate and adaptive immune responses, host–pathogen interactions, and extraintestinal inflammatory signaling (e.g., systemic inflammation, and brain–gut axis communication). Finally, we highlight current challenges and limitations in achieving a causal understanding of microbiota-associated amino acid metabolic alterations in IBD, particularly regarding their origin, spatial distribution, and functional relevance, as well as their potential utility for disease stratification and treatment-response assessment, and discuss how these insights may inform the future development of next-generation biomarkers and precision therapeutic strategies tailored to individual metabolic phenotypes in IBD.

## 1. Introduction

Inflammatory bowel disease (IBD), encompassing Crohn’s disease (CD) and ulcerative colitis (UC), is a chronic, relapsing, and idiopathic inflammatory disorder of the gastrointestinal tract with an incompletely elucidated etiology [1,2]. With accelerating global industrialization and urbanization, the incidence and prevalence of IBD have increased worldwide [3,4], making it a formidable public health challenge. Although the precise pathogenesis remains debated, it is widely accepted that IBD is collectively driven by genetic susceptibility, environmental triggers, aberrant host immune responses, and gut microbiota dysbiosis [5,6]. Current therapies, while effective in many patients, remain constrained by primary non-response, secondary loss of response, and the lack of curative potential, underscoring an urgent need to unravel novel pathogenic mechanisms and identify innovative therapeutic targets [7,8].

Among the emerging frontiers, the metabolic capacity of the gut microbiota has garnered particular attention. The human gastrointestinal tract harbors trillions of microorganisms that constitute a highly complex and dynamic gut microbiota. The extensive enzymatic repertoire of the microbiome endows it with metabolic capabilities fundamentally distinct from those of the host [9,10]. Among microbiota-associated metabolites, short-chain fatty acids (SCFAs) [11] and bile acids [12] have been relatively well characterized in IBD. In contrast, amino acid-derived metabolites have only recently emerged as a distinct and functionally important class of bioactive molecules [13,14]. Notably, amino acid metabolism represents a core functional niche of the gut microbiota. Commensal bacteria can catabolize both dietary and host-derived amino acids to generate an array of bioactive small molecules, for instance, the metabolism of tryptophan yields indoles and their derivatives [15]. Additionally, the microbiota can utilize substrates such as threonine and arginine to synthesize branched-chain amino acids (BCAAs), polyamines, and their derivatives [16,17,18]. These small molecules are increasingly recognized as bioactive signaling molecules that may influence intestinal homeostasis and have been implicated in IBD pathophysiology.

Mounting evidence links alterations in microbiota-associated amino acid metabolism to IBD disease status, activity, progression, and therapeutic response. On the one hand, experimental studies have shown that several amino acid metabolites can exert barrier-protective effects. For example, the tryptophan derivative indole-3-propionic acid (IPA) promotes mucin secretion and upregulates tight junction proteins in intestinal epithelial cells, thereby reinforcing epithelial barrier integrity and facilitating mucosal healing [19]. On the other hand, under IBD pathological conditions, IBD-associated dysbiosis is accompanied by marked alterations in the amino acid metabolome, characterized by a reduced levels of metabolites with reported homeostatic or protective functions [20,21,22] (e.g., indole derivatives) and increased levels of metabolites with potential deleterious or pro-inflammatory activities. This metabolic shift is associated with disrupted host–microbiota interactions in IBD, characterized by both compositional dysbiosis of the gut microbiota and alterations in microbial metabolic functions, and may contribute to the inflammatory intestinal microenvironment.

Given the complex and context-dependent effects of amino acid metabolism on intestinal inflammation, which may range from pro-inflammatory signaling to barrier-protective and immunoregulatory functions, this review aims to critically synthesize recent advances regarding the roles of microbiota-associated amino acids and their clinical associations, mechanistic roles, and translational potential in IBD. We will specifically examine associations between alterations in four representative amino acid metabolic pathways—including tryptophan, aspartate-related, BCAA, and arginine–polyamine metabolism—and disease status, progression, and response to biologic therapies in IBD. Furthermore, we will summarize experimental evidence regarding their roles in intestinal barrier function, immune regulation, and host–microbiota crosstalk. Among these networks, tryptophan metabolism has received the most extensive clinical and mechanistic investigation and therefore receives proportionally greater emphasis in this review. For aspartate-related metabolism, BCAAs, and polyamines, we synthesize available clinical and mechanistic evidence while highlighting the significant knowledge gaps that warrant future investigation. By critically evaluating the latest discoveries in this rapidly evolving field, this review seeks to offer a conceptual framework for understanding microbiota-associated amino acid metabolism in IBD and to highlight promising directions for future biomarker development and metabolically informed therapeutic strategies, particularly those centered on targeted modulation of microbial amino acid metabolism.

***Terminology and classification***

To avoid conflating metabolic association with biosynthetic origin, we use the term “microbiota-associated amino acid metabolites” as an umbrella term throughout this review. These metabolites are broadly classified into three categories: (i) predominantly microbial metabolites, generated mainly through microbial enzymatic pathways, such as indole derivatives (via bacterial tryptophanase) and agmatine (via bacterial arginine decarboxylase); (ii) predominantly host metabolites influenced by the gut microbiota, whose production, availability, or downstream metabolism is modulated by microbial activity, such as kynurenine and serotonin; and (iii) host–microbial co-metabolites, generated through sequential or shared metabolism across microbial and host compartments, such as indoxyl sulfate, which are produced from microbial indole and host hepatic sulfation. These categories indicate the predominant metabolic origin rather than absolute exclusivity, because dietary input and bidirectional host–microbial exchange may also contribute to the measured metabolite pool.

***Literature Search Strategy***

This narrative review is based on a structured literature search conducted in PubMed and Web of Science to identify studies on microbiota-associated amino acid metabolism in IBD, with a focus on clinical metabolomic alterations and experimental mechanisms. Search terms included combinations of “inflammatory bowel disease,” “IBD,” “Crohn’s disease,” and “ulcerative colitis” with “amino acid metabolism,” “metabolomics,” “microbiota,” “intestinal barrier,” “immune regulation,” and “mechanism.” Publications in English from January 2020 to October 2025 were prioritized, with earlier studies retained when they provided foundational evidence. Eligible studies included clinical IBD cohorts with metabolomic or amino acid profiling, and experimental studies investigating mechanisms relevant to intestinal inflammation, barrier function, immunity, or host–microbiota interactions. Studies unrelated to amino acid metabolism or IBD, publications without relevant clinical or mechanistic data, conference abstracts, editorials, commentaries, and non-English articles were excluded. Titles and abstracts were screened, followed by full-text assessment for inclusion. For clinical cohort studies, information on disease subtype, sample matrix, patient characteristics, disease activity, metabolomic platform, direction of metabolite alteration, disease progression, and treatment response was extracted where available.

## 2. Microbiota-Associated Amino Acid Metabolic Networks in IBD

### 2.1. Tryptophan Metabolism

#### 2.1.1. Tryptophan Metabolism Pathway

**Figure 1:**
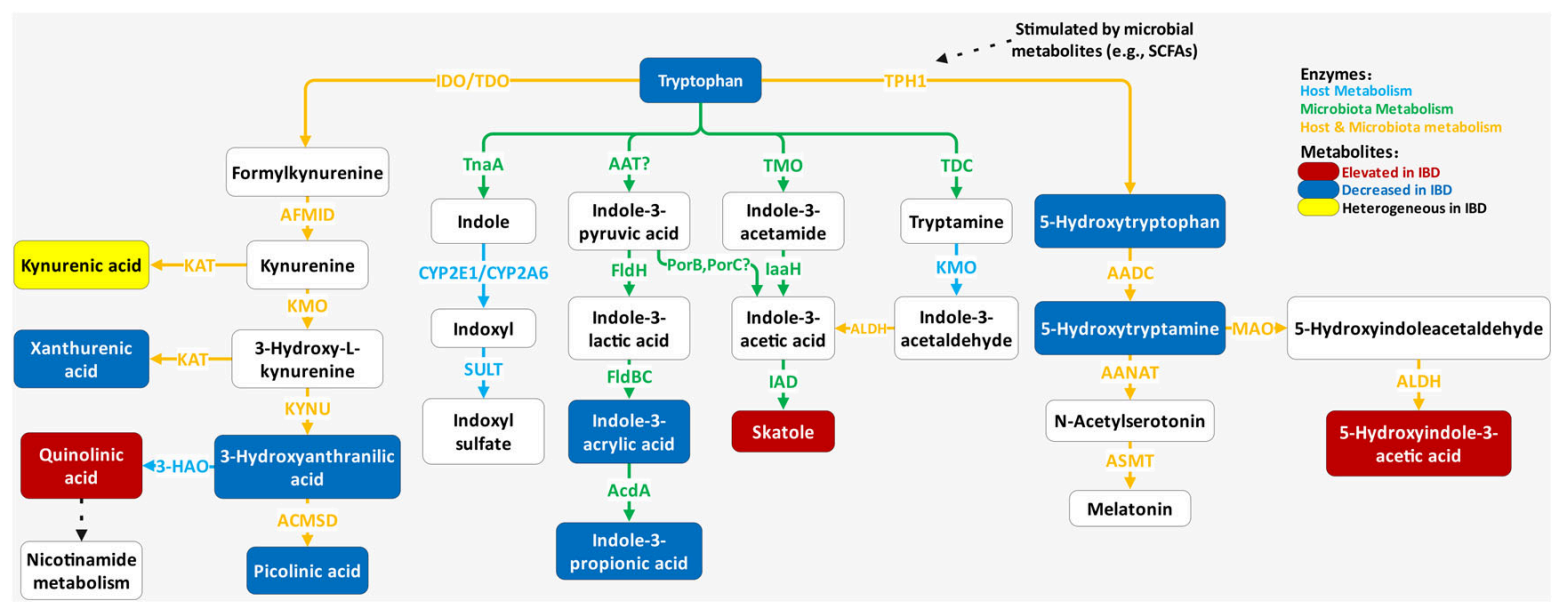
Three Pathways of Tryptophan Metabolism in Host–Microbiota Crosstalk. Tryptophan metabolism is organized into three major interconnected routes: the host-dominated kynurenine pathway, the microbiota-dependent indole pathway, and the enterochromaffin cell-associated serotonin pathway. These pathways generate diverse metabolites, including kynurenine derivatives, indole derivatives, serotonin, 5-HIAA, and melatonin, illustrating the contributions of both host and microbial compartments to tryptophan metabolism. Metabolite nodes with solid-colored boxes indicate those reported to be altered in clinical IBD cohorts. Enzymes shown in blue are of host origin, those in green are of microbial origin, and those in yellow can be found in both the host and microbiota. Full names and EC numbers of the enzymes shown in this figure are listed in Supplementary Table S1.

Within the gut microecosystem, the essential amino acid tryptophan serves as an important metabolic and signaling hub linking the host and the gut microbiota. Its metabolic remodeling is primarily governed by three highly dynamic, intersecting pathways (Figure 1).

***The kynurenine pathway*** represents the major route of host tryptophan consumption influenced by the microbiota through the modulation of indoleamine 2,3-dioxygenase (IDO) activity by microbial signals and inflammatory mediators [23]. Tryptophan is first oxidized to L-formylkynurenine by the rate-limiting enzymes IDO or tryptophan 2,3-dioxygenase (TDO), followed by arylformamidase (AFMID)-mediated hydrolysis to form kynurenine, the central node of this pathway. Subsequently, kynurenine undergoes crucial metabolic shunting driven by a cascade of downstream enzymes: one branch produces kynurenic acid, which exerts cytoprotective effects [24,25,26]; the other main branch involves a series of oxidative cleavages, ultimately yielding the neurotoxic quinolinic acid. Quinolinic acid can be further directed into nicotinamide metabolism, thereby contributing to NAD+ biosynthesis and supporting host cellular energy metabolism and redox homeostasis [27,28].

***The indole pathway*** is predominantly dependent on the unique enzymatic machinery of the gut microbiota. Bacterial tryptophanase (TnaA) directly converts tryptophan into free indole. A fraction of this free indole enters the host bloodstream and reaches the liver, where it is co-metabolized by host enzymes (CYP2E1, CYP2A6, sulfotransferase) into indoxyl sulfate, a host–microbial co-metabolite. In parallel, gut bacteria convert tryptophan into multiple bioactive indole derivatives through distinct enzymatic routes, including indole-3-acetic acid (IAA), indole-3-lactic acid (ILA), indole-3-acrylic acid (IAcrA), indole-3-propionic acid (IPA), tryptamine, and skatole. Several microbiota-associated indole metabolites serve as potent natural ligands for the aryl hydrocarbon receptor (AHR) [29], playing indispensable protective roles in maintaining intestinal epithelial tight junctions and mucosal immune homeostasis.

***The serotonin (5-hydroxytryptamine, 5-HT) pathway*** occurs predominantly in the enterochromaffin (EC) cells of the intestinal mucosa [30,31], and is regulated by gut microbial metabolites that modulate *Tph1* expression—the gene encoding the rate-limiting enzyme tryptophan hydroxylase 1 (TPH1) for peripheral 5-HT synthesis [32]. Although only approximately 1–3% of tryptophan enters the serotonin pathway [33,34], it generates several biologically important signaling molecules. Tryptophan is hydroxylated by TPH1 into 5-hydroxytryptophan, which is then decarboxylated to form the neurotransmitter 5-HT. Free 5-HT undergoes two primary metabolic fates: one part is degraded by monoamine oxidase (MAO) into the inactive 5-hydroxyindoleacetic acid (5-HIAA) and excreted; the remainder is further converted by downstream enzymes into melatonin, a hormone with potent antioxidant and circadian rhythm-regulating properties [35,36].

#### 2.1.2. Clinical Observations in IBD

***Tryptophan and indole derivatives*****:** A comprehensive review of existing cohorts reveals a significant overall reduction in tryptophan levels among IBD patients [20,37,38,39,40], including those after surgery [37] (Supplementary Table S2). Interestingly, in patients receiving biologic therapies, tryptophan levels increased in responders [37]. Furthermore, lower serum levels of IAcrA and IPA have been reported in patients with active IBD [41]. Additionally, skatole (3-methylindole), a microbe-exclusive metabolite, has been reported at higher serum levels in both active and remitting IBD compared to healthy controls [41].

***Kynurenine pathway metabolites*****:** The clinical profile of kynurenic acid in IBD remains heterogeneous across studies and sample matrices. While one study reported higher serum kynurenic acid levels in IBD patients [42], others have reported a decrease in serum kynurenic acid levels [40,43]. In contrast, fecal kynurenic acid levels were found to be elevated in UC patients compared to healthy controls [18]. This discrepancy implies that the functional role of kynurenic acid may shift depending on the local inflammatory milieu or sample origin. Within this metabolic branch, picolinic acid and xanthurenic acid have been reported to display reduced serum levels in IBD patients, whereas quinolinic acid was increased [43]. Another intermediate metabolite, 3-hydroxyanthranilic acid, has been reported at lower serum levels in IBD [43].

***Serotonin pathway metabolites*****:** In the 5-HT pathway, serum 5-hydroxytryptophan is reduced during active IBD compared to healthy controls and patients in remission [41]. Notably, 5-HT itself shows a disease activity-associated increase, with higher serum levels in active IBD than in remission [44]. Conversely, the terminal degradation product of 5-HT, 5-HIAA, is elevated in the serum of IBD patients [43], and higher 5-HIAA levels were associated with an increased risk of disease progression in UC [45]. Given that 5-HIAA is the inactive metabolite of 5-HT, its accumulation may reflect excessive 5-HT turnover rather than enhanced signaling, a distinction relevant to future therapeutic targeting.

### 2.2. Aspartate Metabolism

#### 2.2.1. Aspartate Metabolic Network

**Figure 2:**
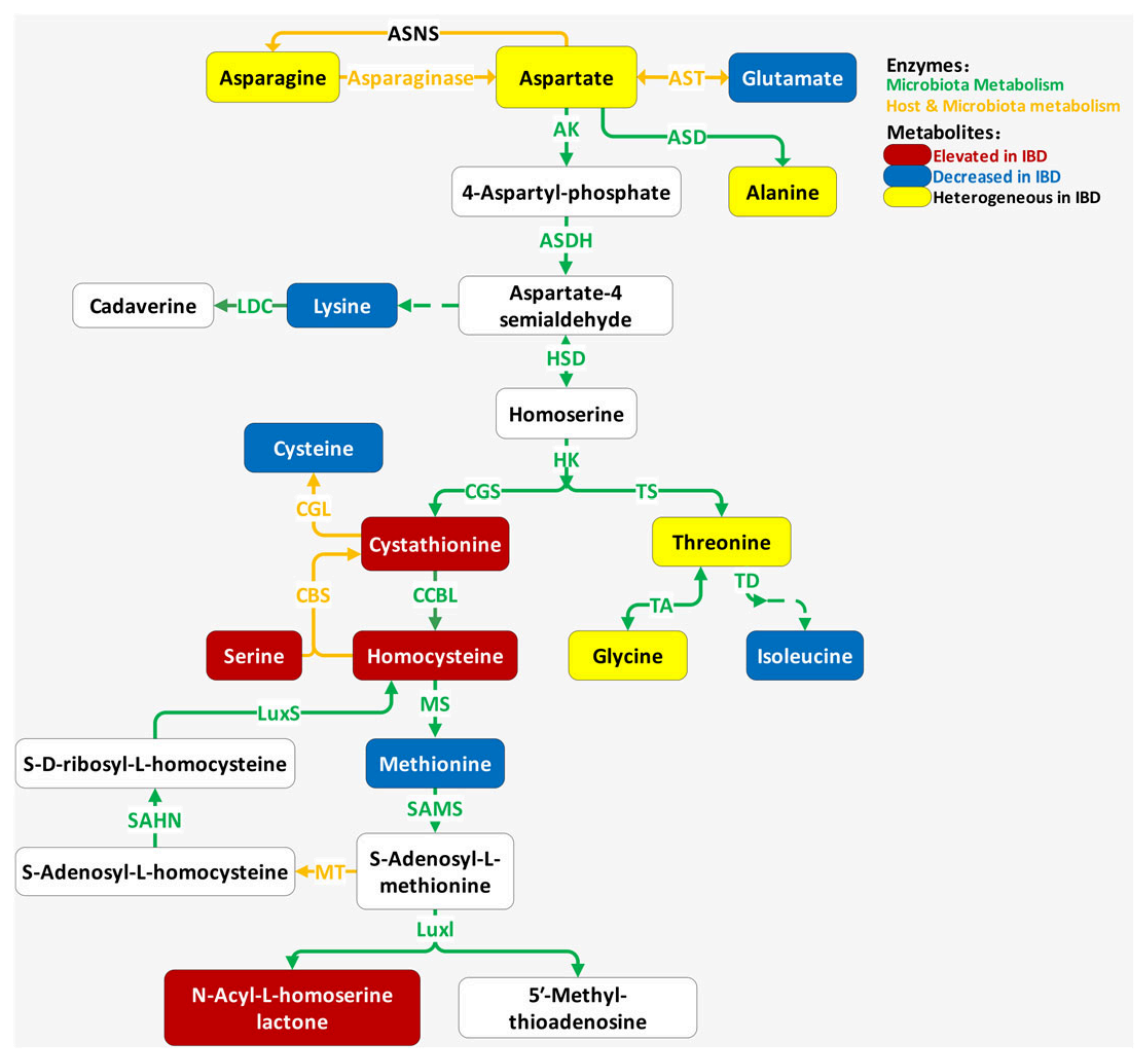
Aspartate as a Metabolic Crossroads in Host–Microbiota Interactions, Linking Amino Acid Biosynthesis, Polyamine Production, and Quorum Sensing. Aspartate-related metabolism connects host energy and nitrogen metabolism with microbial biosynthetic and signaling pathways. Aspartate and glutamate are interconverted through aspartate aminotransferase (AST), whereas aspartate semialdehyde serves as a key branching intermediate toward lysine-derived cadaverine production, homoserine-derived methionine and S-adenosylmethionine (SAM)-dependent quorum sensing (QS), threonine/glycine metabolism, and isoleucine biosynthesis. Metabolite nodes with solid-colored boxes indicate those reported to be altered in clinical IBD cohorts. Enzymes marked in green are derived from the microbiota, and those in yellow are found in both the host and the microbiota. Full names and EC numbers of the enzymes shown in this figure are listed in Supplementary Table S1.

Within the intricate intestinal metabolic network, aspartate and its derivatives act as a biochemical crossroads, bridging host energy metabolism and microbial signal transduction (Figure 2). At the fundamental level of shared host–microbe metabolism, aspartate and glutamate are efficiently interconverted by aspartate aminotransferase (AST). In the hypoxic and hypermetabolic mucosal microenvironment characteristic of IBD, this pair of amino acids serves not only as a crucial anaplerotic substrate to fuel the tricarboxylic acid (TCA) cycle but also as a vital nitrogen and energy pool essential for epithelial proliferation and tissue repair [46,47,48,49]. In addition, aspartate can be decarboxylated to alanine by aspartate 4-decarboxylase (ASD) in some microbial contexts.

However, a marked interspecies division of labor defines the aspartate metabolic network. In microbial metabolism, aspartate semialdehyde acts as the central watershed, directed by distinct enzymes toward either the lysine-to-polyamine pathway or the homoserine-to-quorum-sensing (QS) pathway. The lysine-derived polyamine pathway may influence local inflammatory and redox responses. Gut bacteria convert newly synthesized lysine into free polyamines, primarily cadaverine, a microbial metabolite, via lysine decarboxylase (LDC). These microbe-derived polyamines act as a double-edged sword, regulating mucosal oxidative stress, macrophage infiltration, and local immune responses [50,51]. The homoserine-derived QS pathway can influence the pathogenic remodeling of the IBD microbiome. The microbial metabolite homoserine is converted to microbial methionine via a four-step cascade. Activated by homoserine kinase (HK), the intermediate is sequentially processed by cystathionine γ-synthase (CGS) and cysteine-S-conjugate β-lyase (CBL) into homocysteine. Homocysteine is subsequently methylated by methionine synthase (MS) to form methionine, which is then converted to S-adenosylmethionine (SAM) by S-adenosylmethionine synthetase (SAMS). SAM serves as a precursor for multiple bacterial QS signals. In specific pathobionts (e.g., adherent-invasive *E. coli*), LuxI-type synthases use SAM together with acyl donors to generate a class of QS signaling molecules called N-acyl homoserine lactones (AHLs) [52,53,54]. During IBD pathogenesis, these pathobionts exploit QS signals to orchestrate microbial metabolism and induce pro-inflammatory responses in colonic epithelial cells [55]. Beyond bacterial cell–cell communication, these QS signals may also influence host responses. Notably, a recent study has provided important evidence linking bacterial quorum-sensing molecules to colitis-associated cancer (CAC) in UC patients. Serum levels of short-chain AHL, mainly produced by Gram-negative bacteria, are significantly elevated in UC patients, particularly those with long-standing inflammation for more than 10 years with increased risk of colitis-associated cancer; moreover, systemic administration of C6-scAHL promoted colonic tumorigenesis in a CAC mouse model [54], suggesting a potential link between bacterial QS signaling and inflammation-associated tumorigenesis. In addition, homocysteine, an intermediate of the methionine cycle, and its transsulfuration product, cysteine, are integral to constructing mucosal antioxidant defenses (e.g., glutathione and hydrogen sulfide), and their levels correlate with mucosal oxidative stress and barrier integrity [56,57,58,59,60]. In this pathway, serine condenses with homocysteine via cystathionine β-synthase (CBS) to form cystathionine, which is subsequently cleaved by cystathionine γ-lyase (CGL) to generate cysteine. In a parallel metabolic shunt, homoserine is phosphorylated by homoserine kinase (HK) to form O-phosphohomoserine, which is subsequently converted into threonine via threonine synthase (TS). Serving as a critical bifurcating node, threonine can either be cleaved by threonine aldolase (TA) to yield glycine, or alternatively deaminated by threonine dehydratase (TD) to generate 2-oxobutyric acid. The latter intermediate ultimately cascades through a multi-step pathway to synthesize the branched-chain amino acid isoleucine (see below section). Although these interconversions are primarily microbial, they link homoserine metabolism to a broader network of amino acid availability, with potential implications for the metabolic crosstalk between gut bacteria and the host in IBD.

#### 2.2.2. Clinical Observations in IBD

***TCA cycle-associated pool: Glutamate, aspartate, and asparagine*****:** Glutamate, aspartate, and asparagine function as a vital energy and nitrogen reservoir for the intestinal epithelium, exhibiting distinct alterations in IBD patients. Multiple studies have reported lower serum glutamate levels in IBD patients [38,61] compared to healthy controls, whereas higher glutamate levels have been associated with a lower risk of surgery in patients with CD [45] (Supplementary Table S2). Notably, recent experimental studies showed that L-glutamate supplementation attenuated dextran sulfate sodium (DSS)-induced colitis and that microbiota-associated L-glutamate promoted Foxp3+ Treg expansion through enhanced IL-2 receptor signaling [62,63]. Collectively, these findings support a potential protective role for glutamate in the inflamed gut.

Conversely, the profiles of aspartate and asparagine exhibit pronounced cross-cohort heterogeneity. Lower serum and urinary asparagine levels have been reported in IBD patients [40,61], whereas higher fecal levels have been observed [40]. Aspartate similarly displays cohort-specific divergence in UC, showing a decrease in adult serum [39] but an increase in pediatric feces [40]. Regarding disease progression, higher serum aspartate levels were associated with disease progression in adult patients with CD [45]. Notably, several acetylated and modified derivatives of aspartate and glutamate have also been reported to vary in IBD patients, potentially reflecting alterations in cellular metabolism associated with the inflammatory microenvironment. For instance, N-acetylglutamate is reduced in the feces of pediatric IBD patients [64]. However, higher urine N-acetylaspartate and L-pyroglutamic acid (a cyclized derivative of glutamate) levels have been associated with disease progression in UC [45].

***Microbiota-associated amino acids: Lysine, methionine, and threonine*****:** Lower serum or urine lysine and methionine levels have been reported across several IBD cohorts [39,61,65]. Notably, in cohorts receiving biologic therapies, higher baseline serum levels of lysine and methionine were observed in patients who subsequently responded to biologic therapy [66], suggesting a potential association with treatment response. Furthermore, serum lysine levels negatively correlate with disease progression in UC patients [45]. In contrast to the relatively consistent clinical patterns reported for lysine and methionine, threonine shows substantial heterogeneity in pediatric [38] and adult [61] IBD patients. It is positively correlated with UC progression but negatively correlated with CD progression [40,45], and elevated fecal threonine levels are observed in non-responders to biologic therapy [67]. These divergent clinical associations may reflect differences in disease subtype, sample matrix, or microbial amino acid metabolism; however, whether they arise from altered flux through homoserine-related biosynthetic pathways remains unknown and requires direct mechanistic investigation.

***Redox-sensitive network: Glycine, homocysteine, and cysteine*****:** The clinical profiles of other collateral metabolites derived from the aspartate network are equally complex, yet they are metabolically interconnected. Glycine and cysteine are both essential precursors for glutathione synthesis, a major antioxidant defense system in the intestinal mucosa [68]. Given their involvement in glutathione metabolism, changes in glycine and cysteine levels may be related to altered redox homeostasis in IBD. While some cohorts report lower serum glycine levels in IBD patients [38,61], others [40,69] indicate significant elevation in feces or serum, which positively correlates with disease progression [45]. This discrepancy may reflect disease stage- or compartment-specific differences in glutathione metabolism, although direct flux evidence is lacking. Upstream of cysteine, homocysteine, a sulfur-containing amino acid, is markedly elevated in the serum of UC patients [39]. In parallel, serum cystathionine levels are elevated in IBD patients compared with controls [40]. Homocysteine serves as a substrate for the transsulfuration pathway leading to cysteine, although circulating homocysteine levels do not directly indicate pathway flux. Elevated homocysteine can drive the transsulfuration pathway toward increased cysteine production, yet the subsequent fate of cysteine depends on local redox conditions [57,60]. The behavior of cysteine-related metabolites varies by disease subtype and redox state: the levels of serum sulfur amino acid cysteine is negatively correlated with disease progression and surgical risks in UC patients [45], yet its levels are elevated in the urine of CD patients responding to biologics [70]. In contrast, urine levels of its oxidized dimer, cystine, are reduced in relapsing UC patients [71].

### 2.3. Branched-Chain Amino Acid (BCAA) Metabolism

#### 2.3.1. BCAA Metabolism Pathway in Host and Microbiota

In the gut microecosystem, branched-chain amino acids (BCAAs, comprising leucine, isoleucine, and valine) function as important metabolic substrates and nutrient signals involved in host energy metabolism and mucosal immune homeostasis.

***Host metabolism*****:** BCAAs are essential amino acids that must be obtained from diet. In host tissues, BCAAs are first transaminated by branched-chain aminotransferase (BCAT) to produce branched-chain α-keto acids (BCKAs), which are then irreversibly decarboxylated by the branched-chain α-keto acid dehydrogenase complex (BCKDH). Beyond their role as nitrogen donors and energy substrates, BCAAs—particularly leucine—activate the mechanistic target of rapamycin complex 1 (mTORC1) signaling pathway, thereby promoting protein synthesis, cell growth, and intestinal epithelial renewal [72].

**Figure 3:**
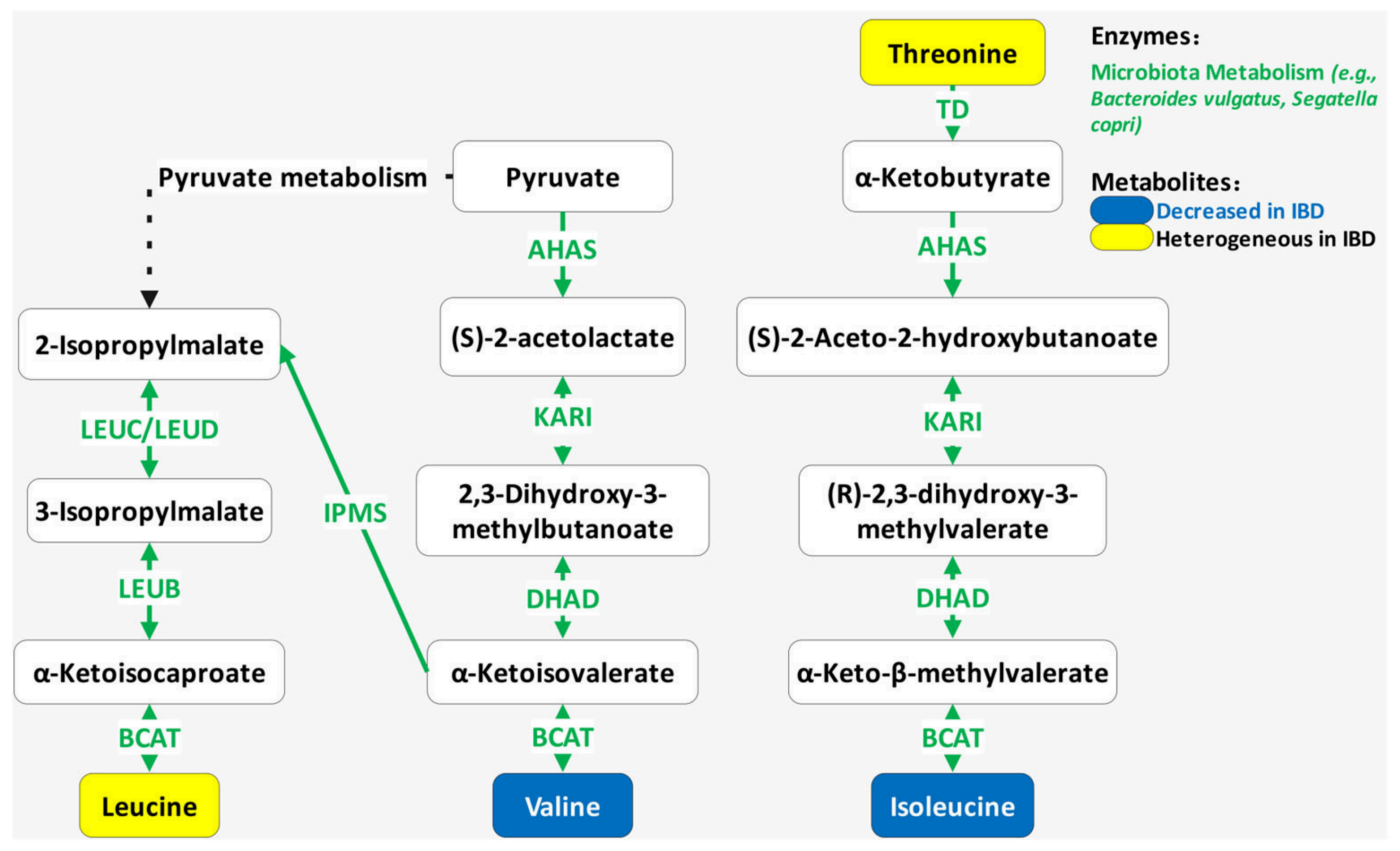
Microbial de novo Biosynthesis of Branched-Chain Amino Acids (BCAAs) in the Gut Microbiota. Many gut microorganisms possess the enzymatic machinery required for de novo BCAA biosynthesis(e.g., *Bacteroides vulgatus* and *Segatella copri*), providing a potential source of locally available BCAAs. Pyruvate-derived intermediates give rise to valine and leucine, whereas threonine-derived α-ketobutyrate is directed toward isoleucine biosynthesis. These biosynthetic routes are catalyzed by key bacterial enzymes, including acetohydroxyacid synthase (AHAS), ketol-acid reductoisomerase (KARI), dihydroxyacid dehydratase (DHAD), 3-isopropylmalate dehydratase (LEUC/LEUD), 3-isopropylmalate dehydrogenase (LEUB), and branched-chain amino acid transaminase (BCAT), potentially contributing to the local intestinal BCAA pool. Metabolite nodes with solid-colored boxes indicate those reported to be altered in clinical IBD cohorts. Enzymes marked in green are derived from the microbiota. Full names and EC numbers of the enzymes shown in this figure are listed in Supplementary Table S1.

***Microbiota metabolism*****:** Unlike mammalian host cells, which lack de novo BCAA biosynthetic pathways, many gut microorganisms possess the enzymatic machinery required for BCAA biosynthesis (Figure 3). Among BCAA-biosynthetic taxa, *Phocaeicola vulgatus* (formerly *Bacteroides vulgatus*) and *Segatella copri* (formerly *Prevotella copri*) have been identified as prominent contributors to microbial BCAA biosynthetic potential [73]. This microbial pathway utilizes threonine and pyruvate as initial substrates. Starting from pyruvate, through a series of reactions, α-ketoisovalerate is generated as a crucial metabolic node; it serves not only as the direct precursor for valine but also branches into the leucine synthesis pathway. Catalyzed by microbial biosynthetic enzymes complexes such as LEUC/LEUD (e.g., produced from bacteria *Phocaeicola vulgatus* and *Segatella copri*), α-ketoisocaproate is formed and ultimately transaminated to yield leucine. Additionally, isoleucine is synthesized from threonine through a related biosynthetic pathway initiated from threonine. Also known as threonine dehydratase, this enzyme initially converts threonine into α-ketobutyrate. This intermediate subsequently undergoes a series of sequential reactions, catalyzed by acetohydroxyacid synthase (AHAS), ketol-acid reductoisomerase (KARI), and dihydroxyacid dehydratase (DHAD), to generate the immediate precursor α-keto-β-methylvalerate. Finally, branched-chain amino acid transaminase (BCAT) completes the synthesis via terminal transamination to yield isoleucine. Thus, gut microbial BCAA biosynthesis may contribute to the local intestinal BCAA pool independently of host dietary intake.

#### 2.3.2. Clinical Observations in IBD

***Reductions in selected circulating BCAAs*****:** Across several clinical cohorts, circulating BCAA levels, particularly isoleucine and valine, have generally been reported to be reduced in IBD (Supplementary Table S2). Compared to healthy controls, serum and urine isoleucine levels are significantly reduced in UC patients [39,61], and serum valine is also diminished in IBD cohorts [61]. This universal contraction of the BCAA pool likely reflects two non-mutually exclusive mechanisms: one is the impaired host absorption secondary to mucosal inflammation, and the other is the compromised microbial BCAA synthesis following dysbiosis [74]. Furthermore, lower BCAA levels correlate with adverse clinical outcomes. In UC patients, serum BCAA levels are negatively correlated with disease progression and surgical risk [45]. In the context of biologic therapy, higher baseline serum levels of BCAA were associated with subsequent response to vedolizumab in UC and overall IBD [66]. Together, these observations indicate that lower circulating isoleucine and valine levels are associated with unfavorable clinical phenotypes; however, this relationship needs to be verified by further experiments.

***Heterogeneity of leucine levels*****:** In contrast to the relatively consistent depletion patterns of isoleucine and valine, the clinical profile of leucine exhibits context-dependent heterogeneity across studies and sample types (Supplementary Table S2). On one hand, while serum leucine levels are decreased in IBD patients compared to healthy subjects [39,61], fecal leucine levels are elevated, as observed in another study [75]. Contradictory findings also exist regarding UC disease severity [45,61]. Such conflicting data for a single amino acid underscore the potential for dynamic shifts in leucine metabolic flux throughout the course of UC. Possible explanations include differences in disease stage (active vs. remission), sample type (serum, urine, feces), medication exposure, or dietary intake, all of which are confounding factors that are often incompletely controlled and warrant rigorous prospective studies.

At the bacterial community functional level based on fecal metagenomics analysis, evidence shows that BCAA transaminase and the superpathway of BCAA biosynthesis were enriched in healthy controls compared to IBD patients [76], and PWY-5103 (L-isoleucine biosynthesis III) and PWY-5104 (L-isoleucine biosynthesis IV) were decreased in IBD cohorts [77]. Interestingly, pathways involved in BCAA biosynthesis were enriched after fecal microbiota transplantation combined with dietary intervention in UC patients [78], indicating that microbial BCAA biosynthesis might be restored by microbiota-targeted interventions, while further validation is needed.

### 2.4. Arginine and Polyamine Metabolism

#### 2.4.1. Arginine and Polyamine Metabolism Pathway

**Figure 4:**
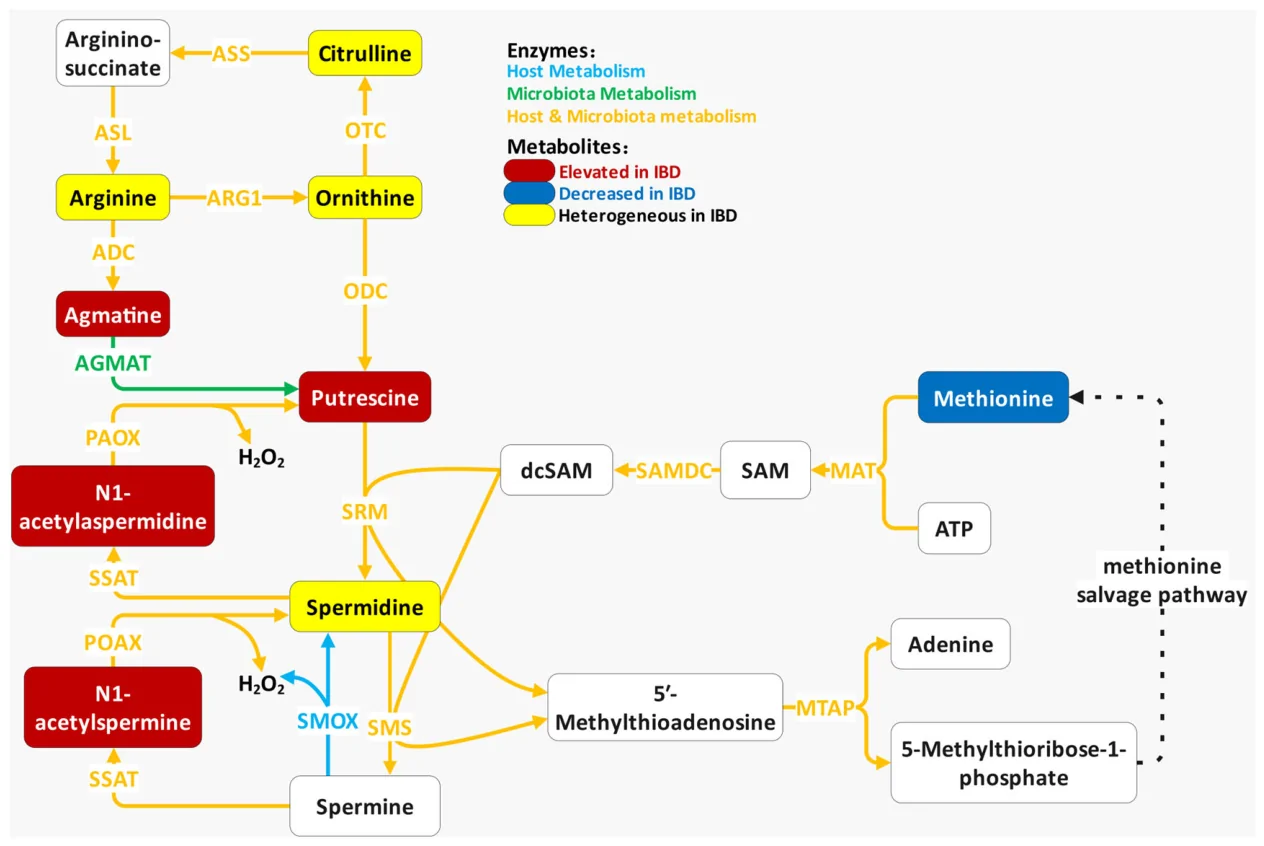
Arginine–Polyamine Metabolism and Polyamine Turnover in the Intestinal Microenvironment. Arginine metabolism contributes to the intestinal polyamine pool through both host and microbial pathways. Host-derived arginine is converted into ornithine, citrulline, putrescine, spermidine, and spermine, whereas microbial arginine decarboxylation generates agmatine. Polyamine acetylation and oxidative degradation produce acetylated derivatives and hydrogen peroxide, linking excessive polyamine turnover to oxidative stress and mucosal injury in IBD. Metabolite nodes with solid-colored boxes indicate those reported to be altered in clinical IBD cohorts. Enzymes marked in blue are derived from the host, those in green from the microbiota, and those in yellow from both the host and microbiota. Full names and EC numbers of the enzymes shown in this figure are listed in Supplementary Table S1.

Within the intestinal metabolic landscape, the conversion of arginine to polyamines represents a highly active biochemical cascade that can be divided into three core phases: substrate conversion, polyamine synthesis, and oxidative degradation (Figure 4).

***Substrate conversion*****:** As the starting point of this pathway, arginine is hydrolyzed by arginase 1 (ARG1) into ornithine and urea. Ornithine can either proceed into polyamine synthesis, or facilitated by ornithine transcarbamylase (OTC), combine with carbamoyl phosphate to form citrulline. Physiologically, citrulline is predominantly synthesized by intestinal epithelial cells and released into the blood; therefore, in clinical and basic research, citrulline levels are often utilized as a gold-standard biomarker for evaluating the number of functional intestinal epithelial cells and the integrity of the intestinal physical barrier [79,80,81]. Recent evidence has extended this paradigm by demonstrating that certain gut commensals, such as *Lactobacillus murinus*, can also synthesize L-citrulline, thereby adding a microbial contribution to the circulating citrulline pool [82].

***Polyamine synthesis*****:** Ornithine is decarboxylated by the rate-limiting enzyme ornithine decarboxylase (ODC) to generate putrescine. The polyamine synthesis network subsequently converges with methionine metabolism: methionine-derived S-adenosylmethionine (SAM) is converted by SAM decarboxylase (SAMDC) into decarboxylated S-adenosylmethionine (dcSAM), which acts as the aminopropyl donor. Catalyzed by spermidine synthase (SRM), putrescine condenses with dcSAM to form spermidine; subsequently, spermine synthase (SMS) facilitates a second addition of dcSAM to finally yield spermine. Physiologically, these polycations (putrescine, spermidine, and spermine) are indispensable for nucleic acid and protein synthesis, exerting potent growth-regulatory effects that promote intestinal epithelial division, proliferation, and mucosal renewal [50,83,84,85,86].

***Oxidative degradation and salvage*****:** In parallel to de novo synthesis, cells employ a stringent mechanism for polyamine degradation and salvage. Spermine and spermidine are initially acetylated by spermidine/spermine N1-acetyltransferase (SSAT) to form N-acetylspermine and N-acetylspermidine, respectively. These acetylated derivatives then serve as substrates for polyamine oxidase (PAOX), undergoing oxidative cleavage to be recycled back into spermidine and putrescine. Crucially, this PAOX-mediated oxidative degradation inevitably generates hydrogen peroxide (H_2_O_2_) [87,88,89,90]. Hyperactivation of this degradation loop yields excessive reactive oxygen species (ROS), thereby promoting local oxidative stress.

#### 2.4.2. Clinical Observations in IBD

***Upstream substrates*****:** As the upstream substrate for polyamine metabolism, arginine shows substantial heterogeneity across diverse IBD cohorts [38,40], and one study indicates alterations in arginine-related metabolism have been associated with subsequent disease progression in CD [45] (Supplementary Table S2). This discrepancy may reflect differences in disease activity, nutritional status, and the relative contribution of host versus microbial arginine utilization. Citrulline, a surrogate marker of functional enterocyte mass, displays similar cohort divergence: it is significantly elevated in both the serum and feces of pediatric IBD patients [40] but markedly reduced in another UC cohort [39]. Nevertheless, lower plasma citrulline levels have been associated with small-bowel involvement in CD and, in some pediatric cohorts, greater disease severity [91,92]. This discrepancy, together with the clinical associations, suggests that a single-time-point measurement may be insufficient to capture dynamic changes in enterocyte mass, and longitudinal sampling may be required. A similar compartment-dependent pattern is also observed for ornithine, the direct precursor for polyamine biosynthesis, which is significantly decreased in the serum of adult UC patients [39], but elevated in the fecal samples from pediatric IBD patients [40,75]. Together, these findings suggest that alterations in the arginine–citrulline–ornithine axis are highly context dependent, shaped by age, disease phenotype and sample type.

***Core polyamines*****:** In contrast to the fluctuating upstream substrates, the overproduction of downstream polyamines and the accumulation of their degradation products demonstrate a much more consistent association with disease exacerbation. Cohort data shows that elevated serum polyamine levels positively correlate with disease progression in UC and surgical risk in CD [45]. Specifically, feces and serum putrescine are significantly enriched in IBD patients [64,93]; the clinical profile of spermidine remains inconsistent, with reports of both significant elevation in [64] and depletion [93] in IBD. The discrepancy between putrescine and spermidine may be explained by differential expression of spermidine synthase or by tissue-specific retention of spermidine. Further studies measuring both tissue and luminal levels are needed.

***Acetylated degradation products*****:** The intermediates of the oxidative degradation loop, N1-acetylspermidine and N1-acetylspermine, were elevated in fecal samples from an IBD cohort [64]. Because acetylated polyamines are products of polyamine turnover, their elevation may indicate altered polyamine metabolism in IBD; however, clinical metabolomic data alone do not establish increased oxidative degradation or consequent mucosal oxidative stress and demand a deeper exploration. Furthermore, agmatine, a specific product of direct microbial arginine utilization, is also elevated in the feces of IBD patients [64]. Even more striking is the high consistency across cohorts regarding the broader suite of acetylated products originating from polyamine degradation. Specifically, N1,N12-diacetylspermine, alongside N-acetylcadaverine (from the lysine-cadaverine bypass) and N-acetylputrescine, are elevated in the feces of IBD patients [64,94]. Furthermore, higher baseline feces N-acetylcadaverine were associated with an increased subsequent risk of IBD-related surgery in CD [45]. Concurrently, higher baseline 4-acetamidobutanoate was associated with an increased risk of subsequent disease progression in UC [45]. These findings suggest that selected acetylated polyamine derivatives may have potential as biomarkers of disease activity or progression, although independent validation is required. Unlike free polyamines, which are subject to rapid cellular uptake and metabolism, acetylated derivatives may better reflect net polyamine turnover [95,96].

### 2.5. Other Amino Acid Metabolites

Beyond the four representative metabolic networks discussed above, fluctuations in several independent free amino acids and their derivatives—though not central to a single dominant pathway—have emerged as important players in IBD pathophysiology. Below, we summarize the diagnostic, prognostic, and therapy-predictive associations of these metabolites with IBD categorized by their functional roles.

#### 2.5.1. Antioxidant-Related Metabolites

As a crucial antioxidant, histidine—a predominantly host metabolite influenced by the gut microbiota—suppresses lipid peroxidation and sustains glutathione synthesis alongside endogenous antioxidant enzymes, directly limiting ROS accumulation during oxidative stress [97,98]. Histidine-related metabolites have also been associated with a lower risk of disease progression [38,39,45,61,65,71], and is significantly elevated in the serum and feces of CD patients who achieve remission with biologic therapy [70]. Its derivative, 1-methylhistidine, is also significantly reduced in the urine of IBD patients [65]. Additionally, specific metabolites relying on gut microbial co-metabolism, including hippurate, a gut-derived phenolic metabolite [43] which has been shown to exert certain antioxidant activity [99,100], and ergothioneine, a potent sulfur-containing antioxidant [41], exhibit consistent depletion in IBD patients. Taurine, a host metabolite influenced by the gut microbiota which regulates cellular osmolarity and oxidative stress [101,102,103,104], is elevated in patient feces [40,75], though conflicting reports note reduced urinary taurine in IBD [65]; prognostically, it is negatively correlated with disease progression and surgical risk in UC [45]. Its precursor, hypotaurine, is also reduced in IBD patients [105]. These findings suggest that alterations in antioxidant-related amino acid metabolism may accompany, and potentially contribute to, disease progression. However, whether these metabolic changes are causal, compensatory, or secondary to chronic inflammation remains unresolved.

#### 2.5.2. Stress-Responsive Metabolites

Concurrently, some amino acids exhibit abnormal accumulation. Serine, a predominantly host metabolite influenced by the gut microbiota, is a non-essential amino acid that serves as a primary donor of one-carbon units via the folate and methionine cycles, thereby supporting the biosynthesis of nucleotides, glutathione, and SAM-processes essential for cell proliferation, redox balance, and epigenetic regulation [106]. Serine is consistently elevated in both serum and feces of pediatric UC cohorts [40], and is positively correlated with adult UC disease progression [45], likely reflecting the massive compensatory demand for metabolic substrates by inflamed tissue during the acute phase. Proline, also a predominantly host metabolite influenced by the gut microbiota, is a conditionally essential amino acid that plays a critical role in collagen biosynthesis, cellular redox reactions, and the regulation of mTOR signaling and gene expression [107]. Proline is elevated in the serum of pediatric IBD patients [40], but its prognostic value exhibits disease-subtype specificity: its elevation positively correlates with disease progression in CD [45] while predicting positive biologic response in CD patients [108]. The elevation in serine and proline in IBD contrasts with the depletion seen for most other amino acids, which may reflect specific metabolic reprogramming of the inflamed mucosa. Future studies should investigate whether these elevations are simply compensatory or actively contribute to fibrosis and stricture formation, particularly in CD.

#### 2.5.3. Aromatic Amino Acids

Despite both being aromatic amino acids, phenylalanine and tyrosine show heterogeneous clinical profiles across IBD cohorts and sample matrices. Depletion of both of them can be observed in IBD [41,61], yet phenylalanine is significantly elevated in the serum and feces of UC patients [40,69]. Prognostically, serum phenylalanine is negatively correlated with disease progression and surgical risk in UC [45], and higher baseline serum phenylalanine levels were associated with subsequent response to vedolizumab [66]. Conversely, serum tyrosine and its sulfated derivative (tyramine O-sulfate, a host–microbial co-metabolite) positively correlate with increased surgical risk in CD and disease progression in UC [45]. Critically, elevated feces tyrosine is associated with non-response to biologic therapies [67]. Furthermore, phenylacetylglutamine, another microbial co-metabolite of these aromatic amino acids, is enriched in the serum of CD patients [109], further substantiating the abnormal metabolic shift in aromatic amino acids under microecological dysbiosis.

**Figure 5:**
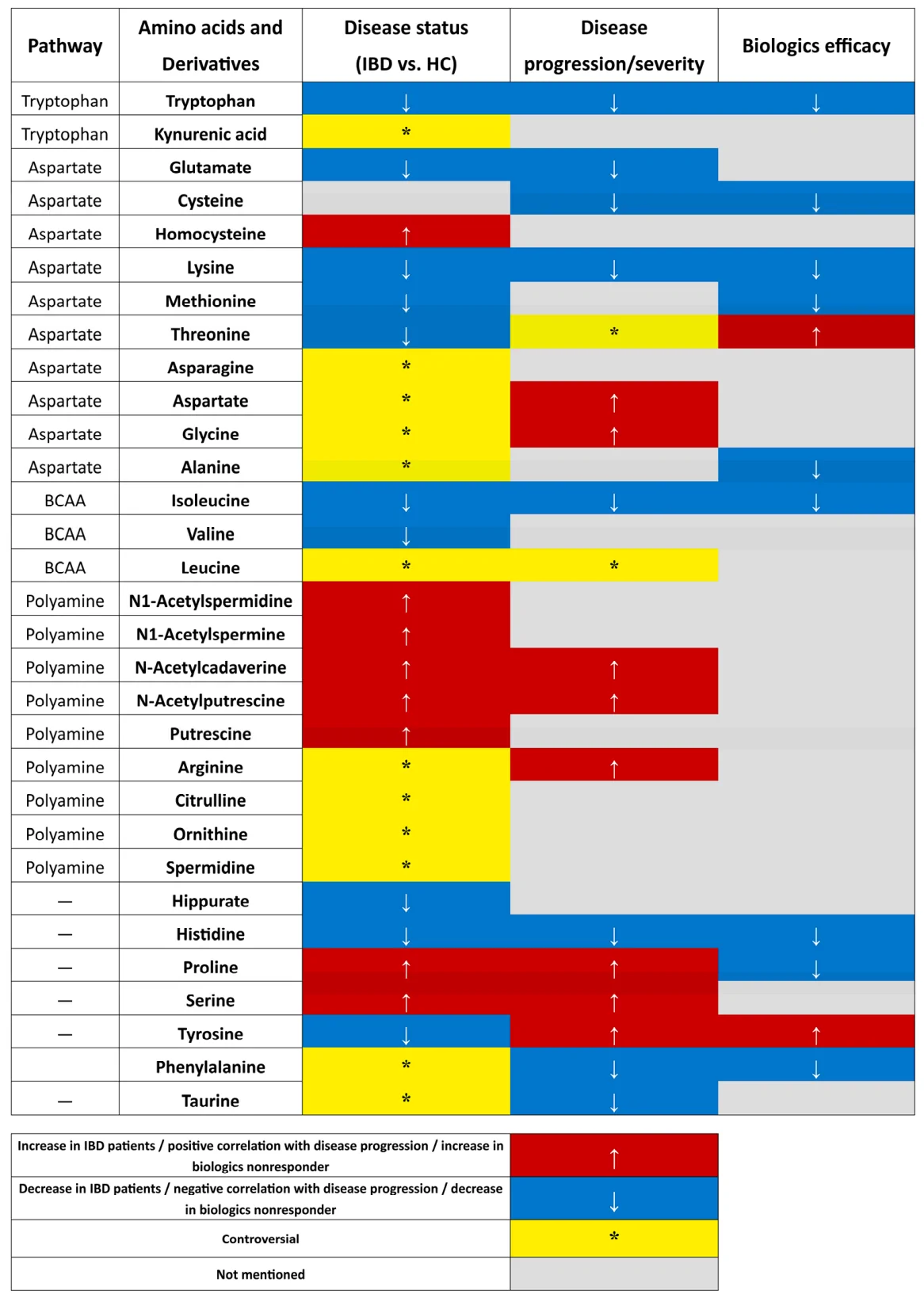
Clinical Associations of Microbiota-associated Amino Acid Metabolites with IBD Diagnosis, Disease Progression, and Biologic Response. This figure summarizes reported alterations in amino acids and their derivatives across IBD clinical contexts, including disease status (IBD vs. HC), disease progression, severity, risk of surgery or relapse, and response to biologic therapies. Relatively consistent patterns include the depletion of tryptophan, glutamate, lysine, methionine, threonine, isoleucine, valine, histidine, phenylalanine, and cysteine, as well as the elevation in serine, proline, putrescine, acetylated polyamine derivatives, and selected disease-associated metabolites. In contrast, kynurenic acid, aspartate, asparagine, glycine, leucine, arginine, citrulline, spermidine, and alanine show heterogeneity. IBD, inflammatory bowel disease; HC, healthy controls. BCAA, branched-chain amino acid.

In summary, clinical metabolomic studies converge on two overarching patterns: reductions in some amino acids metabolites (e.g., tryptophan-derived indoles, glutamate, histidine, lysine, methionine, isoleucine, and valine) and frequent elevations in stress-responsive metabolites (e.g., serine, proline, and acetylated polyamines). Conversely, several hub metabolites, including kynurenic acid, aspartate, glycine, leucine, arginine, and citrulline, exhibit marked heterogeneity, likely reflecting differences in disease subtype, age, sample matrix, and treatment exposure. These trends are summarized in Figure 5, which provides an integrated overview of diagnostic, prognostic, and therapy-predictive associations. The detailed findings from individual clinical cohorts, including metabolite alterations across different sample matrices (e.g., serum, feces, and urine) and IBD subtypes, are compiled in Supplementary Table S2.

## 3. Molecular Mechanisms of Microbiota-Associated Amino Acids in IBD

Following the characterization of the metabolic shifts in amino acids and their derivatives in clinical IBD cohorts, elucidating the specific molecular pathways by which these metabolites regulate intestinal homeostasis is crucial for understanding their pathogenic and protective roles. Current evidence indicates that various classes of amino acids (e.g., tryptophan and its derivatives, aspartate-related metabolites, polyamines, and branched-chain amino acids) alongside their microbial co-metabolites profoundly influence intestinal homeostasis and IBD pathogenesis. They operate through complex molecular networks across multiple dimensions, including the intestinal barrier, mucosal immunity, host–pathogen interactions, and multiple extraintestinal mechanisms.

### 3.1. Regulation of Intestinal Barrier Function

**Figure 6:**
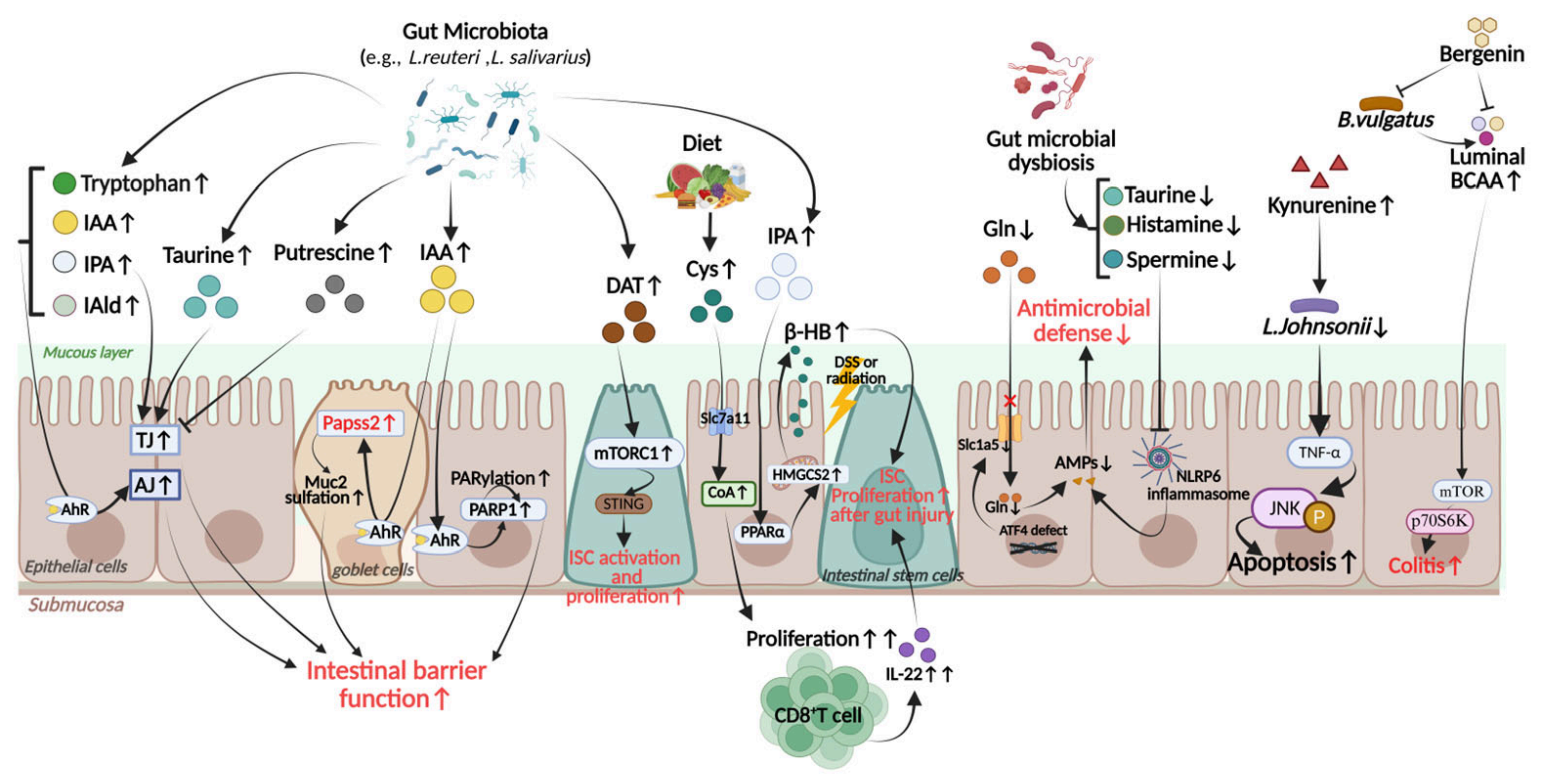
Microbiota-associated Amino Acid Metabolites in Intestinal Barrier Maintenance and Mucosal Repair. Amino acid metabolites regulate multiple layers of intestinal barrier function. Microbial tryptophan metabolites, including IPA and IAA, enhance tight junction integrity, whereas putrescine disrupts tight junctions and taurine stabilizes barrier integrity. IAA promotes mucin sulfation to reinforce the mucus layer. Dietary cystine supports IL-22-dependent intestinal stem cell proliferation. IPA facilitate AHR/PARP1-mediated DNA repair in intestinal epithelial cells. For epithelial immunity, glutamine uptake by intestinal epithelial cells is regulated by activating transcription factor 4 (ATF4) and supports antimicrobial peptide expression, while dysbiosis-associated reductions in taurine, histamine, and spermine may weaken NLRP6 inflammasome-mediated antimicrobial protection. Upward (↑) and downward (↓) arrows indicate increases and decreases, respectively, in the indicated metabolite levels or biological processes. The × symbol indicates blockade or inhibition of the indicated downstream pathway or effect.

The integrity of the intestinal barrier serves as the primary line of defense in maintaining intestinal homeostasis [110]. Various amino acid metabolites play pivotal roles across different processes, including physical barrier maintenance, chemical mucus modification, and epithelial stem cell regeneration (Figure 6).

Microbiota-associated tryptophan metabolites are instrumental in preserving intestinal barrier function. Among them, indole-3-propionic acid (IPA) reduces paracellular permeability by upregulating tight junction proteins (Claudin-1, Occludin, and ZO-1). Simultaneously, it promotes the secretion of MUC2/MUC4 mucins and goblet cell products (TFF3, RELMβ), thereby fortifying the intestinal barrier [19]. As ligands for the aryl hydrocarbon receptor (AHR), microbial tryptophan metabolites such as tryptophol, indole-3-pyruvic acid, indole-3-aldehyde (IAld), and indole-3-acetic acid (IAA) can also preserve the structural integrity of the apical junctional complex and cytoskeletal proteins, including myosin IIA [111]. Furthermore, IAA derived from *Lactobacillus reuteri* activates 3′-phosphoadenosine 5′-phosphosulfate synthase 2 (*Papss2*) transcription and upregulates Papss2 and solute carrier family 35 member B3 (Slc35b3), thereby enhancing the sulfation of intestinal mucins to maintain the mucosal barrier [112]. Beyond its role in mucin sulfation, IAA has also been shown to exert barrier-protective effects through AHR-dependent DNA repair mechanisms. A recent study demonstrated that IAA derived from *Lactobacillus salivarius* activates the AHR-PARP1 axis, wherein activated AHR interacts with poly (ADP-Ribose) polymerase 1 (PARP1), potentiating PARP1 activity and poly (ADP-ribose) polymerization (PARylation). This AHR-PARP1 axis-mediated DNA repair process enhances intestinal barrier function, suppresses inflammation and cell senescence, and ultimately mitigates intestinal aging [113]. Although this finding originates from an intestinal aging model rather than direct IBD studies, it reveals a metabolite-driven regulatory mechanism that may also be relevant to IBD pathophysiology, given that epithelial barrier dysfunction and cellular senescence are increasingly recognized as contributors to intestinal inflammation in IBD.

Under distinct inflammatory conditions, individual amino acids exert targeted regulatory effects in response to specific inflammatory stressors. In the context of IL-13-induced barrier dysfunction, glutamine specifically disrupts the PI3K/Akt signaling pathway to facilitate the restoration of Claudin-1 expression [114]. In addition to these direct signaling mechanisms, microbiota-associated amino acid metabolites can exert either detrimental or beneficial effects on the barrier. A systematic high-throughput screen identified contrasting roles for two microbiota-associated metabolites in regulating intestinal barrier function. Putrescine triggers cytoskeletal contraction and disrupts intestinal tight junction integrity, while taurine acts as a tight junction stabilizer [115]. In mouse models of DSS-induced colitis or *Citrobacter rodentium* infection—both of which impair intestinal barrier integrity and recapitulate key histopathological features of human IBD—putrescine administration exacerbated colon inflammation, increased gut permeability, reduced colon length, and elevated inflammatory cytokines. Notably, co-administration of taurine effectively blocked putrescine-induced tight junction disruption and the exacerbated inflammatory response, underscoring the antagonistic interplay between these two metabolites in modulating intestinal barrier function [115].

Beyond local metabolic imbalances, dysregulated amino acid metabolism driven by immune–micobial crosstalk can also induce epithelial injury. Under severe stressors such as oxaliplatin-induced intestinal toxicity, CD8+ T cell-derived IFNγ overactivates the IDO1 pathway, causing a pathological surge in L-kynurenine. This surge incites severe gut dysbiosis (e.g., the depletion of *Lactobacillus johnsonii*) and triggers a downstream TNF-α/JNK signaling cascade, ultimately resulting in widespread epithelial apoptosis and mucosal damage [116]. Although this intestinal toxicity model which does not directly replicate IBD, it provides useful mechanistic insight into epithelial barrier disruption, a process central to IBD pathogenesis. Notably, elevated kynurenine levels have been consistently observed in IBD patient cohorts (as mentioned in the Section 2.1), suggesting that similar IDO1-driven dysbiosis may contribute to IBD pathogenesis. In parallel, while serum BCAA levels generally decrease in IBD patients as a reflection of global host–microbiota metabolic imbalance, dysbiosis can simultaneously create localized microenvironments with excessive BCAA production. The aberrant proliferation of *Bacteroides vulgatus* elevates luminal BCAA concentrations, which excessively activates the host mTOR/p70S6K signaling pathway, directly aggravating the clinical symptoms of ulcerative colitis. Targeted intervention with bergenin inhibits this commensal strain, thereby lowering BCAA levels and effectively blocking this inflammatory amplification loop [117].

Emerging evidence also highlights the role of amino acid metabolism in intestinal stem cell (ISC) mediated mucosal regeneration. Dietary cystine, a sulfur-containing amino acid, expands the intracellular coenzyme A (CoA) pool upon epithelial uptake. This stimulates CD8αβ+ T cells to secrete IL-22, which in turn drives ISC-mediated mucosal repair following intestinal injury [118]. Furthermore, the tryptophan derivative IPA activates peroxisome proliferator-activated receptor alpha (PPARα) to promote the local synthesis of β-hydroxybutyrate, which acts as a metabolic messenger to stimulate LGR5+ ISCs and accelerate epithelial regeneration [119]. Concurrently, desaminotyrosine (DAT), an aromatic amino acid derivative, promotes ISC proliferation in an mTORC1-dependent manner and engages the STING signaling to help maintain the undifferentiated state of stem cells within the inflammatory microenvironment [120]. Collectively, these mechanisms establish that amino acid metabolism is not merely a source of building blocks but a dynamic regulatory system that integrates microbial signals and stem cell function to maintain gut barrier homeostasis in IBD.

### 3.2. Immunomodulatory Effects of Microbiota-Associated Amino Acids on the Intestinal Mucosa

**Figure 7:**
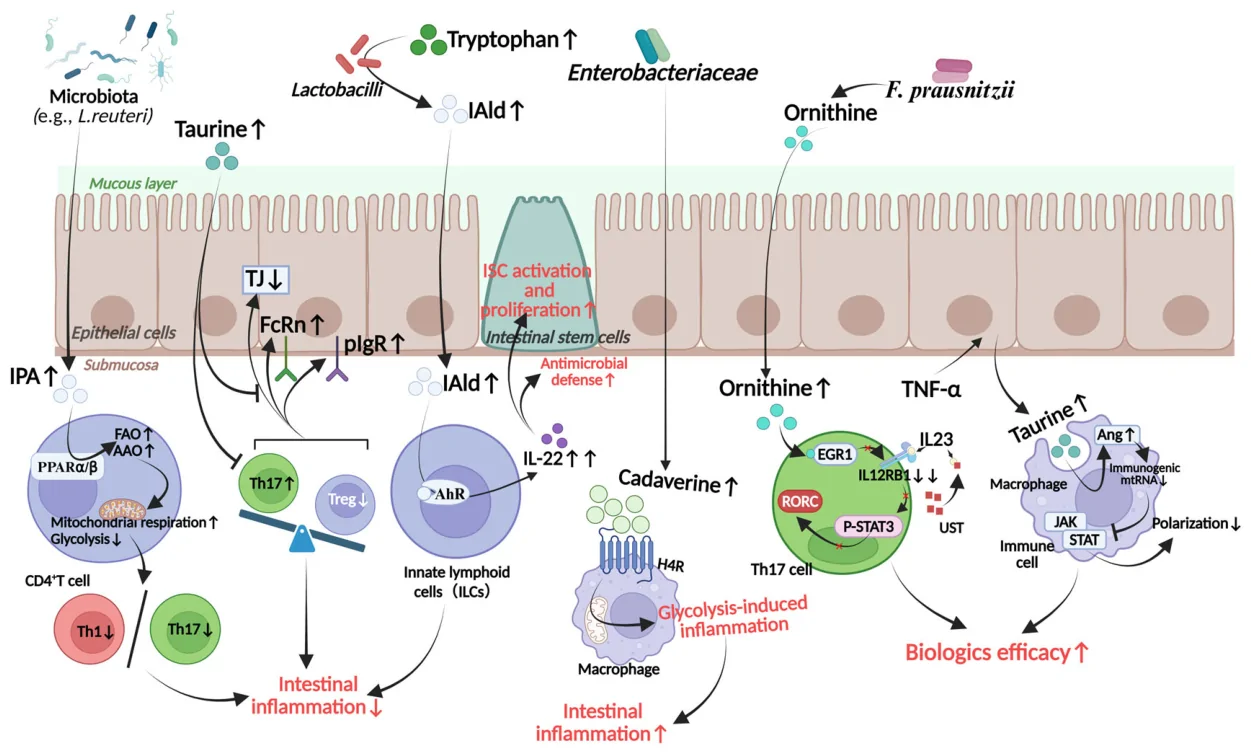
Microbiota-associated Amino Acid Metabolites in Mucosal Immune Regulation. Amino acid metabolites shape intestinal immune responses by targeting multiple cell types. Indole derivatives activate AHR-dependent IL-22 production and promote ISC proliferation; IPA restrains pro-inflammatory CD4+ T-cell differentiation and reduces intestinal inflammation; excessive cadaverine driven by Enterobacteriaceae expansion shifts macrophage metabolism toward glycolysis-driven inflammation; L-ornithine enhances ustekinumab efficacy by suppressing the IL-23, and taurine modulates Th17/Treg balance and macrophage inflammatory signaling to enhance the efficacy of anti-TNF biologic therapies. Upward (↑) and downward (↓) arrows indicate increases and decreases, respectively, in the indicated metabolite levels or biological processes. The × symbol indicates blockade or inhibition of the indicated downstream pathway or effect.

Within the context of intestinal homeostasis, amino acid metabolites serve not merely as nutritional substrates but as potent signaling molecules that orchestrate innate and adaptive immunity. A key example of their role in epithelial innate immunity involves glutamine (Figure 6). Glutamine uptake by intestinal epithelial cells is regulated by activating transcription factor 4 (ATF4). The ablation of ATF4 impairs solute carrier family 1 member 5 (*Slc1a5*) transcription, leading to glutamine deprivation. This drastically diminishes the expression of host antimicrobial peptides, thereby compromising the mucosal antibacterial defense [121]. Broader modulation of epithelial innate immunity is achieved by microbiota-associated metabolites taurine, histamine, and spermine that co-modulate the NLRP6 inflammasome signaling in intestinal epithelial cells. This coordinated activation controls epithelial IL-18 secretion and downstream antimicrobial peptide (AMP) profiles, thereby shaping the host–microbiome interface. Distortion of this balanced pathway by inflammasome deficiency drives dysbiosis, and restoration of this metabolite–inflammasome–AMP axis ameliorates experimental colitis [122]. Beyond epithelial cells, amino acid metabolites shape innate immunity in macrophages (Figure 7). For instance, microbiota-derived cadaverine—the central polyamine in the lysine degradation pathway—regulates macrophage immunometabolism in a context- and concentration-dependent fashion. At baseline, cadaverine is taken up via L-lysine transporters and activates the Nrf2-dependent thioredoxin system, supporting mitochondrial respiration and promoting immunoregulatory macrophage polarization. However, under conditions of elevated cadaverine concentration (driven by *Enterobacteriaceae* expansion), cadaverine signals through the histamine 4 receptor, shifting macrophage metabolism toward glycolysis-driven inflammation and pro-inflammatory functions. Notably, in IBD patients, elevated fecal cadaverine correlates with higher flare risk, underscoring the clinical relevance of this diametric metabolite concentration-dependent effect [123]. In parallel, microbial tryptophan metabolites balance mucosal reactivity via innate lymphoid cells (ILCs). When tryptophan is abundantly available, *Lactobacilli* metabolize it to produce indole-3-aldehyde, which then engages AHR on ILC3s to promote IL-22 transcription. The resulting IL-22-dependent mucosal response enhances epithelial regeneration and antimicrobial defense, providing colonization resistance to pathogens while protecting the mucosa from inflammation-induced damage [124]. Beyond innate immunity, amino acid metabolites also profoundly influence adaptive immunity, particularly through the regulation of T cell differentiation and function. For instance, IPA specifically activates peroxisome proliferator-activated receptor β/δ (PPAR-β/δ), inducing profound metabolic reprogramming in CD4+ T cells. It suppresses glycolysis while enhancing mitochondrial respiration driven by fatty acid oxidation (FAO) and amino acid oxidation (AAO). Consequently, microbiota-derived IPA restricts CD4+ T cell differentiation into pro-inflammatory Th1 and Th17 phenotypes [125]. Another important metabolite in this context is microbial L-ornithine, which has recently emerged as a critical enhancer of ustekinumab (UST) efficacy in Crohn’s disease [126]. A multi-omics study integrating fecal metagenome, metabolome, and host transcriptome data from 85 CD patients revealed that *Faecalibacterium prausnitzii*-derived L-ornithine is significantly elevated in patients who respond favorably to UST therapy. Mechanistically, L-ornithine directly targets the EGR1 transcription factor, suppressing IL-12RB1 transcription and subsequently inhibiting the IL-23-TYK2/STAT3 signaling axis. This pathway disruption destabilizes inflammatory Th17 cells, exhibiting a potent synergistic anti-inflammatory effect when combined with ustekinumab [126]. In addition to these mechanisms, taurine, another amino acid derivative, has been shown to exert immunomodulatory effects through a distinct delivery route. Taurine can be delivered via extracellular vesicles (EVs) derived from *Lactobacillus johnsonii* to orchestrate the Th17/Treg and IgA/IgG composite axes, thereby restoring mucosal immune homeostasis [127]. Mechanistically, *L. johnsonii*-derived EVs reshape the gut microbiota, elevate luminal taurine levels, and directly suppress Th17 differentiation while promoting Treg polarization, ultimately rebalancing the Th17/Treg ratio and reducing immunoglobulin-coated bacteria in colitis [127]. Recent work has further revealed a distinct, epithelium-derived taurine signaling pathway that links intestinal epithelial stress to macrophage-mediated inflammation. Within the intestinal immune microenvironment, TNF-α stimulates epithelial cells to synthesize and release taurine. Upon uptake by macrophages, this epithelium-derived taurine upregulates angiogenin, which specifically degrades stress-induced immunogenic mitochondrial RNA (mtRNA). The targeted clearance of cytosolic mtRNA effectively blunts type I interferon (IFN-I) and downstream JAK-STAT1 signaling, thereby restricting macrophage pro-inflammatory polarization and synergistically enhancing the efficacy of anti-TNF biologic therapies [128]. Collectively, amino acid metabolites orchestrate a multifaceted immunoregulatory network in the intestinal mucosa.

### 3.3. Microbiota-Associated Amino Acid Metabolites Mediate Host–Pathogen Interactions

Amino acid metabolites are central to the biochemical crosstalk between the host and enteric pathogens. This interplay can be conceptualized along a spectrum ranging from host-driven defense to pathogen adaptation and, under pathological conditions, to metabolite-driven pathobiont outgrowth and inflammatory tissue injury. One example of host-driven defense involves dietary tryptophan derivatives. Following microbial metabolism, these derivatives can specifically activate the dopamine D2 receptor (DRD2) in the intestinal epithelium. DRD2 activation downregulates host actin-regulatory proteins, effectively impeding the ability of enterohaemorrhagic *Escherichia coli* (EHEC) to attach and colonize the epithelium via actin pedestals [129]. This illustrates how the host exploits amino acid metabolites to limit pathogen invasion. In turn, enteric pathogens have evolved mechanisms to sense the same metabolites. Enteric pathogens such as EHEC utilize the CpxAR two-component system to sense luminal amino acid derivatives. Upon detecting elevated concentrations of host-derived serotonin or microbial indole, the response regulator CpxR undergoes dephosphorylation, which subsequently silences the expression of the locus of enterocyte effacement (LEE) pathogenicity island; notably, simultaneous elevation in these two signals does not produce an additive inhibitory effect; instead, they antagonize each other, thereby counteracting their individual suppression of pathogen virulence [130]. Thus, amino acid metabolites serve as environmental cues that pathogens use to adjust their virulence programs. Furthermore, certain amino acid metabolites can also be exploited by pathogenic bacteria to gain a competitive advantage in the inflamed gut. A recent study comparing the metabolic profiles of *E. coli* isolates from CD patients, rheumatoid arthritis patients, and healthy individuals revealed that CD-derived *E. coli* strains display a distinct metabolic pattern, characterized by an enhanced capacity to utilize D-serine. This D-serine utilization phenotype depends on the *dsdCXA* gene cluster located within the *argW* locus of the *E. coli* chromosome, which confers a nutritional advantage in both in vitro and in vivo models. Notably, D-serine utilization is significantly more prevalent in CD-derived isolates compared to healthy controls, suggesting that this metabolic trait enables CD-associated *E. coli* to outcompete commensal bacteria under dysbiotic conditions, thereby driving the pathological expansion of Enterobacteriaceae—a hallmark of gut dysbiosis in CD patients [131]. In summary, microbiota-associated amino acid metabolites function as dynamic signaling molecules in host–pathogen interaction, serving both as allies that reinforce host defense and as cues that pathogens exploit to adapt.

### 3.4. Extraintestinal Mechanisms

Amino acid metabolism extends beyond the gut to regulate diverse extraintestinal processes, including systemic inflammation, extraintestinal tissue remodeling, and brain–gut axis communication. One well-characterized pathway linking gut metabolism to systemic inflammation involves phenylacetylglutamine (PAGln). A high-protein diet induces the overgrowth of intestinal *Proteobacteria*, which utilize the phenylpyruvate decarboxylase (PPDC) to generate excessive amounts of PAGln. Upon entering the systemic circulation, PAGln drastically enhances platelet hyperresponsiveness to agonists such as adenosine diphosphate (ADP) and thrombin. This induces CD40 pathway activation, thereby igniting a systemic inflammatory cascade that exacerbates IBD pathology [132]. Beyond the bloodstream, amino acid metabolites can also modulate extraintestinal tissue remodeling, as illustrated by kynurenic acid, a significantly upregulated metabolite in the creeping fat of CD patients, where it correlates with browning marker UCP-1 and inflammatory adipokines. Mechanistically, kynurenic acid activates the GPR35-ERK1/2-PGC-1α signaling pathway to induce white-to-beige transformation of mesenteric adipose tissue, and the resulting browning phenotype promotes M2 macrophage polarization while suppressing inflammation [25], suggesting a potential compensatory anti-inflammatory role for mesenteric browning. Amino acids further act as crucial “long-range signaling” molecules within the brain–gut axis. The kynurenine pathway, a major tryptophan catabolic route activated during intestinal inflammation, generates neuroactive metabolites including kynurenine, kynurenic acid, and quinolinic acid. Kynurenine crosses the blood–brain barrier and is further metabolized within glial cells, where quinolinic acid accumulation correlates with depressive symptoms in IBD, which has been extensively reviewed elsewhere [133,134]. Aromatic amino acids (phenylalanine, tyrosine) contribute to neurotransmitter precursor pools; targeted interventions such as rice protein peptide decrease brain phenylalanine/tyrosine flux, upregulate the downstream metabolites of key neurotransmitters L-DOPA and phenethylamine, and activate the BDNF/TRKB/CREB pathway to ameliorate IBD-associated cognitive impairment and depressive-like behavior [135]. Collectively, these findings establish that amino acid metabolism serve as extraintestinal integrators, linking gut dysbiosis to peripheral inflammation, mesenteric adipose tissue browning and central nervous system dysfunction, thereby opening new avenues for metabolomics-guided therapeutic strategies targeting the gut–extraintestinal axis.

## 4. Challenges and Perspectives

Despite growing recognition of microbiota-associated amino acid metabolites as key signaling molecules in intestinal homeostasis and IBD pathogenesis, several critical challenges remain to be addressed. A major limitation of the current literature—and thus of this review—is the substantial imbalance in mechanistic understanding across different amino acid metabolic networks. While tryptophan metabolism has been extensively characterized at the molecular level, the functional validation of aspartate-related, BCAA, and polyamine pathways remains in its early stages. This disparity should guide future research priorities.

### 4.1. Heterogeneity of Clinical Findings and Technical Barriers

A major obstacle in the field is the considerable inconsistency in reported metabolite alterations across independent cohorts. Metabolites such as leucine, threonine, glycine, taurine, and arginine-related derivatives display considerable heterogeneity among cohorts. This heterogeneity may be attributable to multiple confounding factors, including disease subtype (CD vs. UC), age distribution (pediatric vs. adult), disease activity and duration, anatomical location, sample type (serum, feces, urine, or mucosal tissue), and sampling time point [14,40,136,137]. In addition, differences in dietary patterns, nutritional status, medication exposure (especially antibiotic treatment and biologic therapies), analytical platforms (e.g., LC-MS vs. NMR), and bioinformatic pipelines may further contribute to discrepancies across studies [22,138,139]. Furthermore, most available evidence is derived from cross-sectional observational studies, lacking dynamic characterization of amino acid metabolic alterations. Therefore, standardization of pre-analytical and analytical workflows, as well as the integration of multi-omics data from large, well-phenotyped longitudinal cohorts, will be essential to identify robust, reproducible metabolite signatures. From a translational perspective, future research should also move beyond single-metabolite discovery toward pathway-oriented, multidimensional metabolic signatures. By integrating metabolomics with metagenomics, host transcriptomics, immune phenotyping, and clinical parameters, it may be possible to establish more accurate and robust models for diagnosis, disease stratification, and prognostic prediction. In the setting of biologic therapy, amino acid metabolites may further serve as auxiliary biomarkers for predicting treatment efficacy and identifying patients at risk of non-response, thereby supporting treatment selection, response monitoring, and individualized disease management [70,140,141].

### 4.2. Unresolved Mechanistic Causality

A fundamental limitation of the clinical evidence summarized in this review is that the majority derives from cross-sectional observational metabolomic studies, which can only identify associations between metabolite levels and disease states. It remains unclear whether the observed metabolic shifts in IBD represent primary drivers of inflammation, secondary consequences of tissue injury and dysbiosis, compensatory host responses, or a combination of these processes. Most current studies have focused on tryptophan metabolism and microbiota-associated indole derivatives. By contrast, although BCAAs, aspartate-related metabolites, and polyamine metabolites are repeatedly associated with disease activity, mucosal injury, and therapeutic response in clinical cohorts, their functional targets, upstream and downstream regulatory networks, and causal roles remain insufficiently defined. To move beyond correlative observations, multi-tiered causal validation approaches are warranted. First, Mendelian randomization studies using genetic variants in microbiota-associated amino acid metabolic pathways could help determine whether the observed metabolite–disease associations reflect causal relationships [136]—particularly for metabolites with robust serum-level associations in IBD cohorts, such as tryptophan and histidine. Second, gnotobiotic mouse models colonized with specific metabolite supplementation, or with defined bacterial consortia engineered to produce or lack key metabolites (e.g., indole derivatives IPA or IAA for the tryptophan network) could directly test whether these metabolites actively suppress or aggravate colitis in a controlled in vivo setting. Third, parallel stable isotope tracing [142] (e.g., ^13^C-labeled amino acids) in intestinal organoids derived from IBD patients would allow for quantification of metabolic flux through specific pathways—such as the kynurenine versus indole routes for tryptophan, or polyamine synthesis versus degradation for arginine—under inflammatory versus homeostatic conditions, thereby clarifying whether altered metabolite levels reflect altered production, utilization, or excretion [143]. In addition, spatial metabolomics—an emerging mass spectrometry imaging-based approach that maps the spatial distribution of metabolites directly on tissue sections—could complement these approaches by revealing the precise localization of amino acid metabolites within the intestinal microenvironment [144]. Unlike conventional metabolomics, which averages signals across homogenized tissues, this technique preserves anatomical context and can distinguish whether a metabolite is enriched in the epithelium, lamina propria, or gut lumen—information critical for determining its cellular origin, site of action, and functional relevance in host–microbe interactions. These efforts will provide a mechanistic basis for the development of interventions targeting key metabolic pathways.

### 4.3. Host–Microbiota Metabolic Interactions

Despite mounting evidence linking specific gut microbes to altered amino acid profiles [22,138,145,146], the specific microbial taxa, functional genes, metabolic enzymes, and regulatory networks governing microbial amino acid metabolism remain poorly understood. How dysbiosis shifts the balance between protective (e.g., IPA, IAA) and deleterious (e.g., kynurenine, putrescine, acetylated polyamines) metabolites is largely unexplored. Moreover, bidirectional crosstalk between host and microbial metabolism—such as competition for dietary amino acids, reciprocal regulation of enzyme expression, and metabolite-driven modulation of host immune pathways—requires systematic investigation. Integrating metabolomics with metagenomics, transcriptomics, and immune phenotyping in well-defined IBD cohorts as well as utilization of germ-free animal models and defined bacterial colonization will help decode these complex interactions.

A particularly critical but underexplored dimension of host–microbiota metabolic interactions is competition for amino acid substrates within the intestinal lumen [147]. The gut represents a competitive ecosystem where host epithelial cells and gut bacteria compete for the same dietary and host-derived amino acids. This competition has profound implications for interpreting metabolomic data: a decrease in serum tryptophan, for instance, could reflect increased microbial consumption, decreased host absorption due to epithelial damage, or enhanced host catabolism via IDO1 activation—each with distinct biological meanings and therapeutic implications. These findings highlight the importance of metabolic flux and compartmentalization which distinguishes luminal, mucosal, and systemic metabolite pools. A metabolite elevated in feces may not correlate with its concentration in the mucosal microenvironment where it exerts biological effects. Likewise, a metabolite depleted in serum may be actively produced and utilized within the gut lumen. The apparent “paradox” of systemic BCAA depletion versus local gut BCAA accumulation [117] serves as a case in point: serum and fecal levels reflect distinct biological processes—systemic availability versus local microbial production—and their coexistence is not contradictory but rather highlights the multifaceted nature of host–microbial metabolic interactions in IBD. Future studies should employ multi-compartment sampling (serum, feces, urine, and mucosal biopsies) combined with stable isotope tracing to track amino acid flux from diet to microbiota to host. This approach would help resolve whether the observed metabolic alterations reflect true deficiencies, active consumption, or redistribution between compartments—a distinction critical for designing rational metabolic interventions.

### 4.4. Therapeutic Potential of Targeting Amino Acid Metabolism

Despite the challenges, amino acid metabolic networks offer promising translational opportunities for IBD management. Potential strategies include precision nutritional approaches (e.g., supplementation with specific amino acids or their precursors [148,149]), probiotics [150,151] or engineered bacterial strains designed to restore protective metabolite production [152,153], and small-molecule modulators targeting rate-limiting enzymes in either host or microbial pathways. For instance, enhancing indole derivative production via a tryptophan-rich diet [111,154] or AHR agonists [112,155,156,157], reducing putrescine or acetylated polyamine levels by inhibiting ODC/SSAT [158,159], or using natural compounds like bergenin to suppress pathogenic *B. vulgatus* expansion [117] represent plausible strategies. However, it should be noted that these strategies remain largely preclinical, and clinical translation faces substantial hurdles, including safety concerns for AHR agonists and metabolic enzyme inhibitors in vivo, and delivery and colonization challenges for probiotics and engineered bacteria. Moreover, the net biological effect of any amino acid metabolite intervention is likely context-dependent, depending on concentration, tissue compartment, disease stage, inflammatory milieu, and baseline gut microbial ecology [160]. For example, polyamines such as spermidine have been shown to exert dose-dependent protective effects in colitis models by promoting anti-inflammatory macrophage polarization and preserving epithelial barrier integrity [161], whereas excessive polyamine degradation can generate oxidative stress and contribute to mucosal injury [90]. This underscores that therapeutic strategies should not assume a uniform “beneficial” or “harmful” designation for any given metabolite, but rather consider its dynamic interplay with concentration, site of action, and the underlying inflammatory state. Therefore, future therapeutic strategies should move beyond targeting single metabolites in isolation and instead adopt a pathway-oriented, personalized approach that integrates the patient’s metabolomic profiling, microbiota composition, inflammatory status, and treatment stage. In this regard, deeper characterization of microbiota-associated amino acid metabolic networks may help shift IBD therapy from conventional anti-inflammatory and immunosuppressive approaches toward a precision regulatory framework centered on host–metabolism–microbiota interactions.

Despite these promising avenues, several translational barriers must be critically considered. First, delivering targeted metabolites to the inflamed intestinal mucosa without systemic toxicity remains a major challenge. Oral amino acid supplementation, for example, is subject to extensive first-pass microbial metabolism in the proximal gut, potentially limiting the fraction that reaches distal inflammatory sites [162]. Second, microbial adaptation and resistance pose a non-trivial risk: engineered bacterial strains or probiotics may lose their metabolic capacity over time or be outcompeted by resident pathobionts. Third, the interconnectedness of metabolic networks complicates selective modulation. Interventions aimed at enhancing one protective metabolite (e.g., IPA via tryptophan supplementation) may inadvertently shunt flux toward competing pathways (e.g., kynurenine or serotonin), with unpredictable net effects [163]. Fourth, the field currently lacks well-validated pharmacodynamic biomarkers for monitoring therapeutic efficacy; without such tools, clinical trials risk insufficient statistical power or misinterpretation of negative findings. These challenges highlight the necessity of rigorous preclinical and early-phase clinical studies that explicitly account for the above-mentioned translational hurdles.

In conclusion, the evidence synthesized in this review supports the notion that microbiota-associated amino acid metabolites are not merely passive indicators of dysbiosis, but rather active participants in, and regulators of, intestinal inflammation and tissue repair. The field is gradually moving beyond observational associations toward a deeper understanding of causality, and unraveling the complexities of microbiota-associated amino acid metabolism in IBD will require coordinated efforts in large-scale longitudinal cohort studies, multi-omics integration, mechanistic dissection, and interventional trials. Addressing these challenges will not only advance our fundamental understanding of host–microbe interactions but also pave the way for next-generation diagnostics and therapeutics tailored to the metabolic phenotype of individual IBD patients.

## Data Availability

Data sharing is not applicable for this manuscript as no new data has been created or analyzed in this study.

## Supplementary Materials

The following supporting information can be downloaded at: https://www.mdpi.com/article/10.3390/microorganisms14092108/s1. Supplementary Table S1: Abbreviations, Full Names, and EC Numbers Used in the Figures; Supplementary Table S2: Summary of clinical studies reporting microbiota-associated amino acids and related metabolite alterations in IBD across different clinical contexts and sample matrices, including disease characteristics, patient age, direction of association, adjustment or matching variables, metabolomic platform, and reference.
