# Supplementary material for: Microbiota-Associated Amino Acid Metabolites in Inflammatory Bowel Disease: Emerging Key Players in the Host–Microbe Interface
Source: Microorganisms. 2026 Sep 20;14(9):2108. doi: 10.3390/microorganisms14092108 (PMC13609578; doi:10.3390/microorganisms14092108)
Supplement: Supplementary file 1 [file microorganisms-14-02108-s001.zip › Supplementary_Table_S1 .pdf]

Supplementary Table S1. Abbreviations, Full Names, and EC Numbers Used in the Figures

Abbreviation	Full name	EC number
**Figure 1**		
3-HAO	3-hydroxyanthranilic acid 3,4-dioxygenase	1.13.11.6
AADC	aromatic L-amino acid decarboxylase	4.1.1.28
AANAT	arylalkylamine N-acetyltransferase	2.3.1.87
AAT	aromatic amino acid aminotransferase	2.6.1.57
AcdA	acyl-CoA dehydrogenase	1.3.8.7
ACMSD	2-amino-3-carboxymuconate-6-semialdehyde decarboxylase	4.1.1.45
AFMID	arylformamidase	3.5.1.9
ALDH	aldehyde dehydrogenase	1.2.1.3
ASMT	acetylserotonin O-methyltransferase	2.1.1.4
CYP2A6	cytochrome P450 Family 2 Subfamily A Member 6	-
CYP2E1	cytochrome P450 family 2 subfamily E member 1	1.14.14.-
FldBC	phenyllactate dehydratase	4.2.1.167
FldH	phenyllactate dehydrogenase	1.1.1.110
IaaH	indole-3-acetamide hydrolase	3.5.1.-
IAD	indoleacetate decarboxylase	4.1.1.115
IDO	indoleamine 2,3-dioxygenase	1.13.11.52
KAT	kynurenine aminotransferase	2.6.1.7
KMO	kynurenine 3-monooxygenase	1.14.13.9
KYNU	kynureninase	3.7.1.3
MAO	monoamine oxidase	1.4.3.4
PorB,C	pyruvate:ferredoxin oxidoreductases B and C	1.2.7.8
SULTs	sulfotransferase	-
TDC	tryptophan decarboxylase	4.1.1.105
TDO	tryptophan 2,3-dioxygenase	1.13.11.11
TMO	tryptophan 2-Monooxygenase	1.13.12.3
TnaA	tryptophanase	4.1.99.1
TPH1	tryptophan Hydroxylase 1	1.14.16.4
**Figure 2**		
AK	aspartate kinase	2.7.2.4
ASD	aspartate 4-decarboxylase	4.1.1.12
ASDH	aspartatesemialdehyde dehydrogenase	1.2.1.11
ASNS	asparagine synthase	6.3.5.4
AST	aspartate Transaminase	2.6.1.1
CBS	cystathionine beta-synthase	4.2.1.22
CCBL	cysteine-S-conjugate β-lyase	4.4.1.13
CGL	cystathionine gamma-lyase	4.4.1.1
CGS	plant cystathionine γ-synthase	2.5.1.160
GNMT	glycine N-methyltransferase	2.1.1.20
HK	homoserine kinase	2.7.1.39
HSD	homoserine dehydrogenase	1.1.1.3
LDC	lysine decarboxylase	4.1.1.18
Luxl	acyl-homoserine-lactone synthase	2.3.1.184
LuxS	S-ribosylhomocysteine lyase	4.4.1.21

Abbreviation	Full name	EC number
MS	methionine synthase	2.5.1.13
SAHN	adenosylhomocysteine nucleosidase	3.2.2.9
SAMS	S-adenosylmethionine synthetase	-
TA	threonine aldolase	4.1.2.48
TD	threonine dehydratase	4.3.1.19
TS	threonine synthase	4.2.3.1
**Figure 3**		
AHAS	acetohydroxyacid synthase	2.2.1.6
BCAA	branched-chain amino acid	-
BCAT	branched - chain amino acid transaminase	2.6.1.42
DHAD	dihydroxyacid dehydratase	4.2.1.9
IPMS	isopropylmalate synthase	2.3.3.13
KARI	ketol-acid reductoisomerase	1.1.1.86
LEUB	3-isopropylmalate dehydrogenase	1.1.1.85
LEUC/LEUD	3-isopropylmalate dehydratase large/small subunit	4.2.1.33
TD	threonine dehydratase	4.3.1.19
**Figure 4**		
ADC	arginine decarboxylase	4.1.1.19
AGMAT	agmatinase	3.5.3.11
ARG1	arginase 1	3.5.3.1
ASL	argininosuccinate lyase	4.3.2.1
ASS	argininosuccinate synthetase	6.3.4.5
dcSAM	decarboxylated S-adenosylmethionine	-
MAT	methionine adenosyltransferase	2.5.1.6
MTAP	methylthioadenosine phosphorylase	2.4.2.28
ODC	ornithine decarboxylase	4.1.1.17
OTC	ornithine transcarbamylase	2.1.3.3
PAOX	polyamine oxidase	1.5.3.17
SAM	S-adenosylmethionine	-
SAMDC	S-adenosylmethionine decarboxylase	4.1.1.50
SMOX	spermine oxidase	1.5.3.16
SMS	spermine synthase	2.5.1.22
SRM	spermidine synthase	2.5.1.16
SSAT	spermidine/spermine N1-acetyltransferase	2.3.1.57
**Figure 6**		
ADP	adenosine diphosphate	-
AHR	aryl hydrocarbon receptor	-
AJ	apical junction	-
AMPs	antimicrobial peptides	-
BCAA	branched-chain amino acid	-
CoA	coenzyme A	-
Cys	cysteine	-
DAT	desaminotyrosine	-
Gln	glutamine	-
HP Diet	high-protein diet	-
IAA	indole-3-acetic acid	-

Abbreviation	Full name	EC number
IAld	indole-3-aldehyde	-
IPA	indole-3-propionic acid	-
ISC	intestinal stem cell	-
JNK	c-Jun N-terminal kinase	-
mTOR	mechanistic target of rapamycin	-
NLRP6	NOD-like receptor family pyrin domain-containing 6	-
PAGln	phenylacetylglutamine	-
PAPSS2	3′-phosphoadenosine 5′-phosphosulfate (PAPS) synthase 2	-
PARP1	poly(ADP-ribose) polymerase 1	-
PARylation	poly(ADP-ribosyl)ation	-
PPDC	phenylpyruvate decarboxylase	-
SLC1A5	solute carrier family 1 member 5	-
Slc7a11	solute carrier family 7 member 11	-
TJ	tight junction	-
TNF-α	tumor necrosis factor-alpha	-
**Figure 7**		
AAO	amino acid oxidation	-
AHR	aryl hydrocarbon receptor	-
EGR1	early growth response 1	-
FAO	fatty acid oxidation	-
ILC	innate lymphoid cell	-
IPA	indole-3-propionic acid	-
JAK-STAT1	Janus kinase–signal transducer and activator of transcription 1	-
mtRNA	mitochondrial RNA	-
PPAR-β/δ	peroxisome proliferator-activated receptor β/δ	-
STAT3	signal transducer and activator of transcription 3	-
Th1	T helper 1 cell	-
Th17	T helper 17 cell	-
Treg	regulatory T cell	-
UST	ustekinumab	-
